# Combinatorial hydrogels with biochemical gradients for screening 3D cellular microenvironments

**DOI:** 10.1038/s41467-018-03021-5

**Published:** 2018-02-09

**Authors:** Sebastián L. Vega, Mi Y. Kwon, Kwang Hoon Song, Chao Wang, Robert L. Mauck, Lin Han, Jason A. Burdick

**Affiliations:** 1 Department of Bioengineering, University of Pennsylvania, Philadelphia, PA 19104 USA; 2 School of Biomedical Engineering, Science and Health Systems, Drexel University, Philadelphia, PA 19104 USA; 3 Department of Orthopaedic Surgery, McKay Orthopaedic Research Laboratory, Perelman School of Medicine, University of Pennsylvania, Philadelphia, PA 19104 USA

## Abstract

3D microenvironmental parameters control cell behavior, but can be challenging to investigate over a wide range of conditions. Here, a combinatorial hydrogel platform is developed that uses light-mediated thiol-norbornene chemistry to encapsulate cells within hydrogels with biochemical gradients made by spatially varied light exposure. Specifically, mesenchymal stem cells are photoencapsulated in norbornene-modified hyaluronic acid hydrogels functionalized with gradients (0–5 mM) of peptides that mimic cell-cell or cell-matrix interactions, either as single or orthogonal gradients. Chondrogenesis varied spatially in these hydrogels based on the local biochemical formulation, as indicated by Sox9 and aggrecan expression levels. From 100 combinations investigated, discrete hydrogels are formulated and early gene expression and long-term cartilage-specific matrix production are assayed and found to be consistent with screening predictions. This platform is a scalable, high-throughput technique that enables the screening of the effects of multiple biochemical signals on 3D cell behavior.

## Abstract (web summary)

Tools to investigate a wide range of 3D microenvironmental parameters are important for understanding and controlling cell fate. Here, the authors develop hydrogels with orthogonal biochemical gradients and use this screening system to identify microenvironments that induce mesenchymal stem cell chondrogenesis.

## Introduction

The extracellular matrix (ECM) presents cells with a complex and dynamic microenvironment featuring an assortment of physical and biochemical cues^1^. The extent to which these ECM signals regulate cell behavior largely depends upon dimensionality. For example, stark differences in chondrogenic gene expression of articular chondrocytes in 2D versus 3D cultures have been reported^2^, and the effects of dimensionality on phenotype across other cell types have been observed as well^3,4^. This critical role of dimensionality in cellular interpretation of microenvironmental signals presents the need for 3D culture environments. However, one major challenge is efficiently investigating the influence of large numbers of microenvironmental parameters on cell behavior; thus, screening platforms that can be used to assess cell behavior in 3D are needed.

Indeed, the mixing of two polymer solutions to create single gradients of elastic modulus^5^ and opposite gradients of growth factors^6^ have been explored for 3D culture. However, this technique produces large hydrogels with coarse gradients and is unable to produce miniaturized hydrogels necessary for combinatorial screening. This motivates the need for alternative approaches to introduce multiple material signals in physiological ranges that can be used to study cell behavior. To increase the number of conditions that can be analyzed in 3D, there has been a recent increase in the development of high-throughput platforms amenable to 3D cell culture^7–12^. For example, Ranga and coworkers developed a microarray system to expose hydrogel-encapsulated mouse embryonic cells to over 1,000 unique combinations of physical and biochemical signals to study their combinatorial influence on self-renewal^7^. Likewise, Dolatshahi-Pirouz et al. used a robotic microarray spotter to develop miniaturized hydrogels with encapsulated mesenchymal stem cells (MSCs), exposing them to various ECM proteins and media formulations^8^. By analyzing individual cell-laden hydrogels, they identified specific combinations of ECM proteins that enhanced the expression of osteogenic markers, and showed that these formulations were scalable to larger hydrogel constructs. Most recently, Sharma and colleagues^9^ used microarrays to spatially control the presentation of multiple peptides on nanofibrous materials. While these strategies allowed for the rapid investigation of numerous biochemical and physical parameters on cell behavior, they required advanced robotic nanomixers and spotters for implementation, which limits the accessibility of such techniques to many researchers.

The photopatterning of hydrogels is an alternative and potentially simpler approach that can be used to introduce various signals into hydrogels to probe 3D cell-biomaterial interactions^13^. However, many hydrogel systems amenable for photopatterning use the same light-induced reaction (e.g., methacrylates and photoinitiator) to both form and pattern hydrogels^14^, which couples the patterns to changes in crosslink density. The use of click reactions has emerged as a technique to form hydrogels amenable to subsequent light-mediated modifications, while at the same time obviating this concern^15^. For example, Alge and coworkers implemented a tetrazine-norbornene inverse electron demand Diels-Alder reaction chemistry to form poly(ethylene glycol) hydrogels that support 3D cell culture, which could be subsequently modified with different peptides^16^. Similarly, Gramlich et al. demonstrated the use of thiol-norbornene chemistry to create hyaluronic acid (HA) hydrogels amenable to subsequent modifications with mono-thiolated peptides^17^. Such chemistry has been used to introduce continuous gradients of biochemical and physical cues into hydrogels^18,19^; however, these systems have been limited to 2D interactions, where cells are seeded atop hydrogels after patterning has occurred. These hydrogels have not been explored for 3D encapsulation, and the selected chemistry has limited the range of signals that can be introduced. In the context of tissue development, N-cadherin, a transmembrane protein that mediates cell-cell adhesion, is considered important for chondrogenesis to occur^20^, and the His-Ala-Val (HAV) motif in the first extracellular domain of N-cadherin acts to mimic this interaction^21,22^. Furthermore, the Arg-Gly-Asp (RGD) sequence from fibronectin has been described extensively to mediate cell-matrix interactions^23^. Most recently, in 2D cultures on HA surfaces, Cosgrove et al. reported that these two biochemical inputs can ‘compete’ with one another to alter MSC mechanosensing and fate decisions in the context of changing matrix stiffness^24^.

Here, thiol-norbornene light-mediated reactions between HA macromers functionalized with norbornene groups (NorHA) and di-thiol crosslinkers are used to create hydrogels and encapsulate cells. The unreacted norbornenes in the hydrogel are then reacted with mono-thiolated peptides using light exposure controlled with a sliding opaque mask. This approach is used to introduce either single or multiple gradients of peptides to cells in a 3D environment, where cells are easily imaged for quantifiable outcome measures. To demonstrate the utility of this system, human MSCs are photoencapsulated in NorHA hydrogels and subsequently patterned with peptides that represent cell-cell (e.g., N-cadherin) and cell-matrix (e.g., fibronectin) signals to investigate how their level and synergy influence MSC chondrogenesis. Thus, a platform is reported here that could have widespread applicability to the investigation of many cell types and their surrounding 3D microenvironment.

## Results

### Fabrication of hydrogels with continuous peptide gradients

**Fig. 1:**
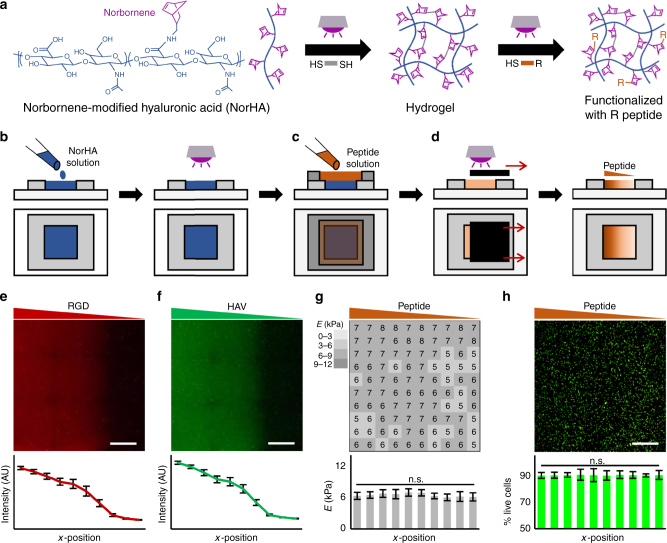
Scheme for fabricating NorHA hydrogels with single peptide gradients and characterization. **a** Multi-step thiol-norbornene UV light-mediated reaction between norbornene-modified hyaluronic acid (NorHA) and di-thiols (DTT) to form a hydrogel and then to subsequently modify with mono-thiolated peptides. Schematic of fabrication process where, **b** NorHA hydrogels were first formed on 5 × 5 × 0.5 mm^3^ molds with UV light exposure, **c** incubated with 5 mM mono-thiolated peptide solution, and **d** peptide gradients introduced with an opaque sliding mask to control the extent of light-mediated reaction between peptides and norbornenes in the hydrogel. Images and quantification of signal intensity of **e** rhodamine-labeled RGD (GCGYGRGDSPG) or **f** fluorescein-labeled HAV (HAVDIGGGC) peptide gradients. **g** Atomic force microscopy (AFM) of the peptide-modified hydrogel presented as a heat map and quantification across the *x*-position (*n* = 10 measurements per zone). **h** Images and quantification of mesenchymal stem cell (MSC) viability within NorHA hydrogels with peptide gradient (RGD shown) (*n* > 1000 cells per zone). Error bars represent standard error around the mean (s.e.m.); scale bars: 1 mm; n.s., no significance between groups

NorHA was synthesized from its tetrabutylammonium salt (HA-TBA) and ~57% of the disaccharide repeat units were modified with pendant norbornenes, as confirmed by ^1^H NMR (Supplementary Figure 1). HA was selected as the backbone for the hydrogels due to its high versatility with respect to chemical functionality^25^ and its ability to support chondrogenesis in 3D hydrogels^26^. To create NorHA hydrogels, norbornenes underwent a thiol-ene reaction with a di-thiol crosslinker (dithiothreitol, DTT), and unreacted norbornenes in the network served as reactive groups for subsequent reactions with thiolated peptides (Fig. 1a). To modify NorHA hydrogels with peptides in a spatially-controlled manner, NorHA hydrogels were first prepared on a 5 × 5 × 0.5 mm^3^ mold by adding a NorHA solution comprised of 4 wt% NorHA macromer, crosslinker (DTT, at a level of 20% theoretical reaction of norbornenes with thiols), and 0.05 wt% photoinitiator (I2959), followed by irradiation with UV light (10 mW/cm^2^) for 10 min (Fig. 1b). NorHA hydrogels were then incubated in a 5 mM peptide solution in PBS for 30 min (Fig. 1c), and the extent of peptide tethering was controlled spatially by exposing hydrogels to UV light (5 mW/cm^2^) for varying amounts of time (0–60 s) with an opaque sliding mask that was moved across the hydrogel at a constant rate (83.3 µm/s) (Fig. 1d). After washing, the resultant hydrogel contained a gradient of tethered peptide.

To visualize peptide gradients, either rhodamine-labeled mono-thiolated RGD (Fig. 1e) or fluorescein-labeled mono-thiolated HAV (Fig. 1f) peptides were included in the peptide incubation solution prior to patterning. In both cases, fluorescence intensity was altered through the duration of light exposure, with higher intensities resulting from more light exposure. Irradiation (5 mW/cm^2^) of whole hydrogels incubated with fluorescent peptides for various times indicated that 60 s was adequate for maximum coupling (Supplementary Figure 2). In addition, fluorescent peptide solutions experienced no loss in fluorescent signal for up to 100 s of irradiation (10 mW/cm^2^) (Supplementary Figure 3), which was beyond the irradiation time and intensity used to couple peptide gradients. To demonstrate that functionalizing hydrogels with biochemical gradients did not affect mechanics, atomic force microscopy (AFM) was used to indent biofunctionalized hydrogels at 100 positions (10 by 10 array). For each position, force-extension curves were fitted to a Hertz indentation model to attain the local indentation moduli. The resulting data was then used to generate a spatial heat map of indentation moduli (Fig. 1g), which showed minimal changes in modulus across the hydrogel and no trend corresponding to peptide tethering. The viability of encapsulated MSCs was also evaluated in the combinatorial hydrogels. MSCs were photoencapsulated in NorHA hydrogels, exposed to HAV or RGD peptides and gradients generated, and evaluated for viability across 10 discrete regions. Viability was high ( > 90%) in all regions after 7 days in culture and there were no trends corresponding to peptide tethering (RGD gradient shown in Fig. 1h; HAV gradient shown in Supplementary Figure 4). Taken together, these results demonstrate the development of a cytocompatible platform for the introduction of gradients of peptides to cells in 3D without changing the mechanical environment.

### MSC chondrogenesis is controlled by peptide gradients

The NorHA hydrogel platform was used to encapsulate MSCs with gradients of RGD or HAV (0–5 mM) to probe their influence on MSC fate. Previous work has shown that low weight percent HA hydrogels provide adequate nutrient diffusivity, are cell compatible, and favor chondrogenesis^27^. Thus, the NorHA macromer and DTT crosslinker concentrations used here were chosen based on these findings, while providing excess pendant norbornenes for subsequent biochemical peptide modifications. The covalently-crosslinked hydrogels also expose encapsulated cells to a 3D environment in which cells retain a rounded morphology that further favors chondrogenesis. HAV and RGD peptides were chosen due to their potential influence on chondrogenesis, based on their importance during development. Within the developing limb bud, mesenchymal condensation occurs, where cells interact with each other via N-cadherin, a cell surface protein that can be mimicked to an extent by HAV peptides^20^. Subsequently, cells begin to spread apart and secrete their own matrix which includes fibronectin, an adhesion molecule that can be mimicked to an extent by RGD^28^. Indeed, hydrogels functionalized with the HAV and RGD motifs have been shown to regulate chondrogenesis of MSCs encapsulated in hydrogels. For example, Bian et al. reported that the conjugation of HAV to HA hydrogels enhanced the expression of chondrogenic genes and cartilage-specific matrix production of human MSCs^22^. In contrast, Connelly and coworkers showed that functionalizing alginate hydrogels with RGD had an inhibitory effect on the chondrogenic potential of bovine MSCs^29^.

To assess cellular outcomes, fluorescent staining and confocal microscopy were used to evaluate single cell behavior across the hydrogels. Although there are multiple markers that are suitable for evaluating chondrogenesis, in this study two markers were chosen based on their well-established role during distinct stages of chondrogenesis and their relationship to one another. The transcription factor Sox9 was selected as a marker for chondrogenesis after 1 day, due to the relevance of Sox9 during development and its consideration as the master regulator for chondrogenesis^30^. Sox9 activation can also be quantified by its presence within cell nuclei, which makes it particularly appealing as a marker for cell screening studies; methods for imaging cells within hydrogels and quantifying the nuclear localization of several markers have been recently reported^21,31^. Aggrecan production after 7 days was selected as a downstream marker of chondrogenesis due to its relevance in cartilage matrix^32^. Specifically, it is the key component of cartilage tissue that provides articular cartilage with its compressive properties and hydraulic permeability^33,34^. Furthermore, its expression is driven directly by the Sox trio containing Sox9 via promoter binding^35^. These two markers can be readily imaged via microscopy and provide spatial evidence of chondrogenesis based on local peptide presentation. Even though Sox9 and aggrecan were used here to screen for chondrogenic environments, the markers and timing can be readily altered based on the material and cell system of interest. In this study, all cultures were performed in the presence of TGF-β3, a soluble factor known to enhance chondrogenesis.

**Fig. 2:**
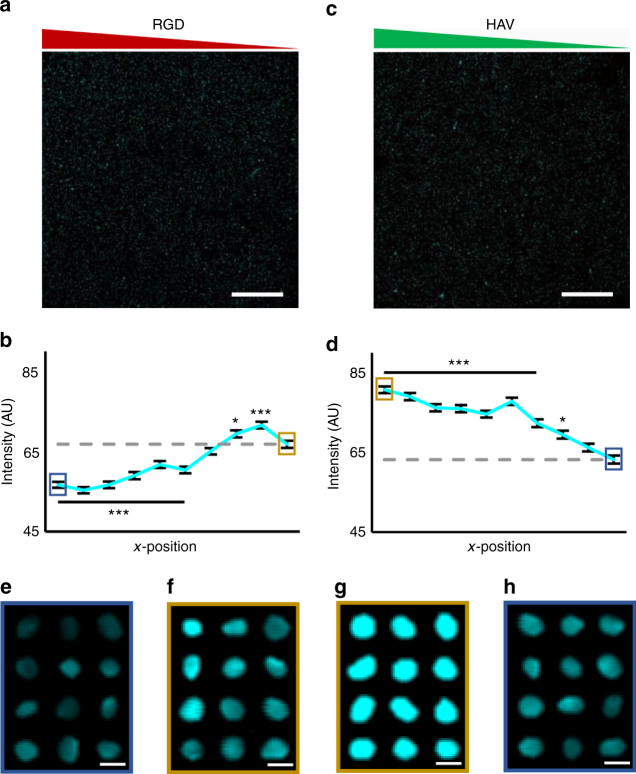
Effects of HAV and RGD gradients on transcription factor Sox9 expression. Maximum projection images and quantification across 10 regions of nuclear Sox9 fluorescence (aqua) for gradients of **a**, **b** RGD and **c**, **d** HAV peptides after 1 day. Representative maximum projection images of nuclei stained for Sox9 in **e** high or **f** low RGD regions and **g** high or **h** low HAV regions. Generally, higher nuclear Sox9 was observed with decreasing RGD and for increasing HAV. Error bars represent standard error around the mean (s.e.m.); *n* > 300 cells per zone, ***p < 0.001, *p < 0.05 compared to lowest peptide region (gray dashed line). Scale bars: **a**, **c** 1 mm, **e**–**h** 10 µm

Using confocal imaging after 1 day of culture, whole hydrogels were imaged and divided into 10 vertical regions prior to analysis. For each region, 3D nuclear masks of individual cells were used to isolate total Sox9 intensity within corresponding nuclei. Sox9 fluorescence was then normalized to nuclear volume, and the average values across cells were reported along the peptide gradient. Analysis of nuclear Sox9 fluorescence in hydrogels with an RGD gradient indicated that RGD has a negative dose-dependent effect on nuclear Sox9 fluorescence (Fig. 2a, b). In contrast, there was a positive relationship between nuclear Sox9 fluorescence and HAV peptide concentration (Fig. 2c, d). Representative maximum projections of Sox9-stained nuclei of MSCs exposed to high and low RGD concentration regions show an increase in fluorescence with lower RGD (Fig. 2e, f); in contrast, representative maximum projections of Sox9-stained nuclei of MSCs exposed to high and low HAV concentration regions show a decrease in fluorescence with lower HAV (Fig. 2g, h).

**Fig. 3:**
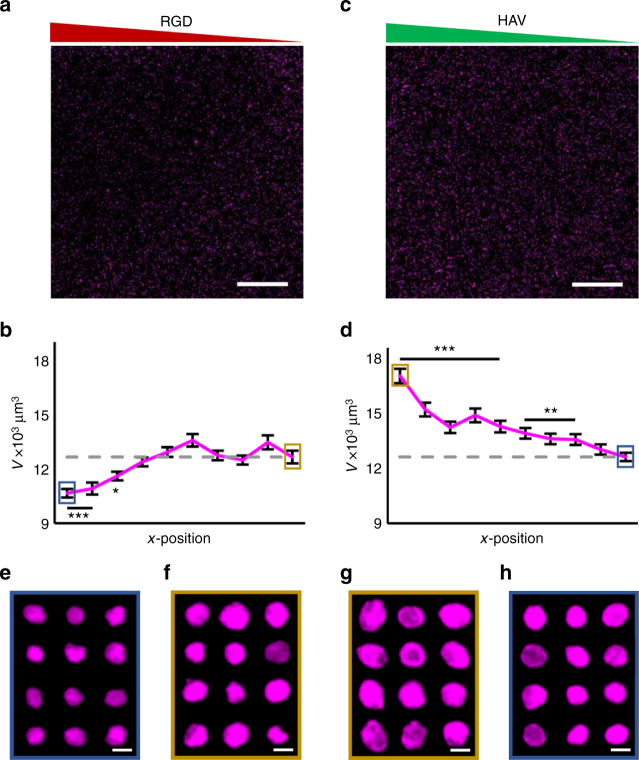
Effects of HAV and RGD gradients on aggrecan synthesis. Maximum projection images and quantification across 10 regions of aggrecan fluorescence (magenta) for gradients of **a**, **b** RGD and **c**, **d** HAV peptides after 7 days. Representative maximum projection images of aggrecan in **e** high or **f** low RGD regions and **g** high or **h** low HAV regions. Generally, larger aggrecan volumes were observed with decreasing RGD and for increasing HAV peptide levels. Error bars represent standard error around the mean (s.e.m.); *n* > 300 cells per zone, ***p < 0.001, **p < 0.01, *p < 0.05 compared to lowest peptide region (gray dashed line). Scale bars: **a**, **c** 1 mm, **e**–**h** 25 µm

MSC-laden NorHA hydrogels with either RGD or HAV biochemical gradients were cultured for 7 days and samples were immunostained for aggrecan (Fig. 3). Using confocal imaging, whole hydrogels were imaged and divided into 10 vertical regions prior to analysis. 3D masks from the aggrecan channel of individual cells were then used to calculate the volume of synthesized aggrecan for each cell. The average values of cellular aggrecan were then reported along the hydrogel’s concentration gradient. Analysis of synthesized aggrecan showed a negative trend between RGD peptide concentration and aggrecan volume (Fig. 3a, b). As observed with nuclear Sox9 fluorescence, there was also a positive trend between synthesized aggrecan and HAV peptide concentration (Fig. 3c, d). Taken together, these data indicate that the RGD peptide alone has a negative influence on chondrogenic markers Sox9 and aggrecan, whereas the HAV motif alone has a positive effect on chondrogenic markers. Importantly, the results indicate that the platform is simple to fabricate and cellular outcomes are easily imaged to assess microenvironmental instruction of cell behavior.

### Fabrication of hydrogels with orthogonal peptide gradients

**Fig. 4:**
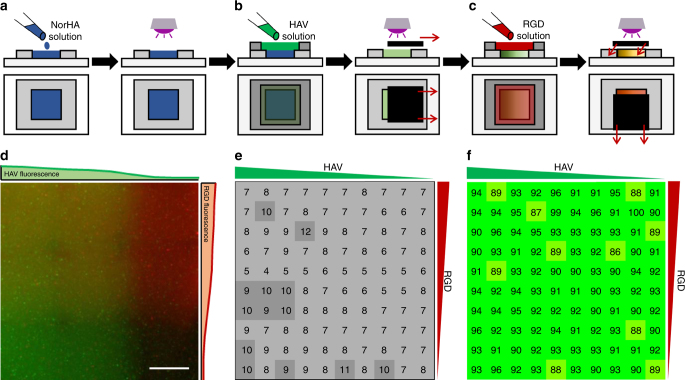
Scheme for fabricating orthogonal biochemical gradients and characterization. **a** Norbornene-modified hyaluronic acid (NorHA) hydrogels were prepared on 5 × 5 × 0.5 mm^3^ molds via a thiol-norbornene UV light-mediated reaction between NorHA and di-thiol (DTT) crosslinker. **b** Hydrogels were then incubated in 5 mM mono-thiolated HAV peptide solution for 30 min and a gradient was generated with an opaque horizontal sliding mask to control the extent of light-mediated reaction between HAV peptides and norbornenes in the hydrogel. After washing, the hydrogel was then (**c**) incubated in 5 mM mono-thiolated RGD peptide solution for 30 min and an opaque sliding mask in the vertical direction was used to introduce a secondary orthogonal gradient. **d** Images of rhodamine-labeled RGD and fluorescein-labeled HAV orthogonal gradients, including quantified intensity profiles on each side. **e** Atomic force microscopy (AFM) indention moduli and **f** encapsulated mesenchymal stem cell (MSC) viability (after 7 days of culture) heat maps of hydrogels with orthogonal peptide gradients. Scale bar: 1 mm

Although the use of single gradients provides a plethora of information regarding the influence of peptide concentration on cell behavior in 3D, the incorporation of orthogonal peptide gradients allows the generation of information related to synergy between peptides across large concentrations. Thus, following the creation of a hydrogel and then introduction of a single biochemical gradient, a second perpendicular gradient can be introduced based on the reaction of thiolated peptides to remaining norbornenes (Fig. 4a–c). To assess the orthogonal peptide gradients, a whole hydrogel exposed to the scheme was imaged, showing the presence of both peptides (Fig. 4d). To quantify fluorescence, image stacks were separated into RGD (horizontal gradient) and HAV (vertical gradient) channels, and fluorescence was measured on 10 vertical and horizontal regions, respectively (Supplementary Figure 5). AFM was performed across the hydrogel as a 10 by 10 grid to ensure that mechanics did not change within the hydrogel based on peptide modifications. No substantial differences in indentation moduli were observed across the hydrogel (Fig. 4e). The viability of encapsulated MSCs was also evaluated in the combinatorial hydrogels. MSCs were photoencapsulated in NorHA hydrogels, exposed to orthogonal HAV and RGD biochemical gradients, imaged, divided into 100 discrete regions, and evaluated for viability (Fig. 4f). Viability was determined to be at least 85% for all regions after 7 days in culture. Thus, orthogonal gradients were generated using a cytocompatible approach where various combinations of peptides are introduced without changes in hydrogel mechanics.

### MSC chondrogenesis varies within combinatorial hydrogels

**Fig. 5:**
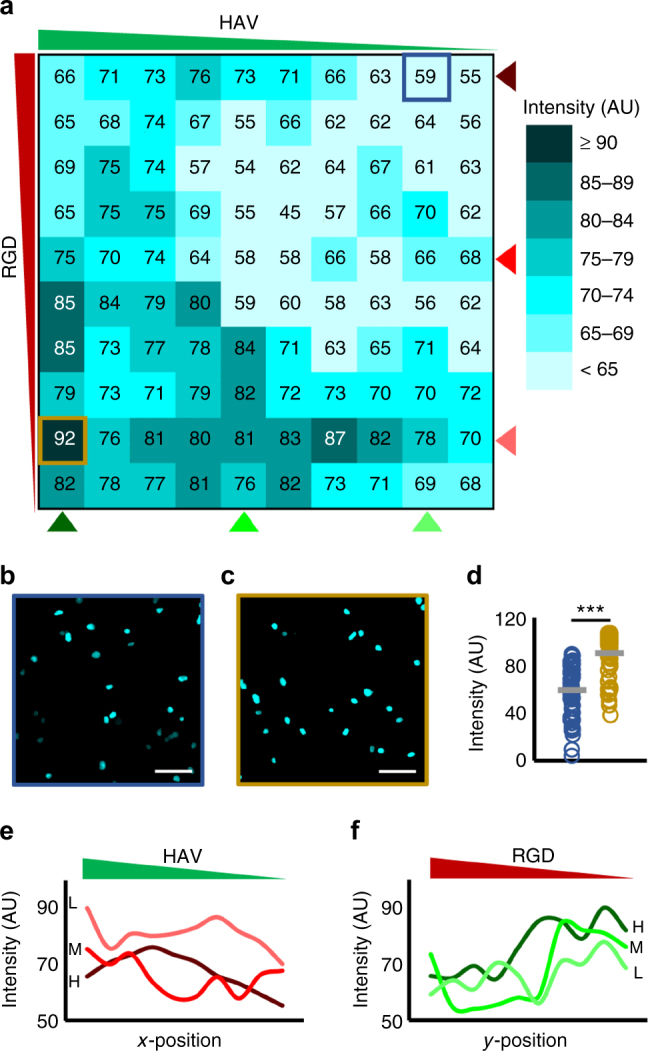
Effects of orthogonal HAV and RGD gradients on transcription factor Sox9 expression. **a** Heat map of average intensities of Sox9 fluorescence across 100 bins (10 by 10 array) for cultures of mesenchymal stem cells (MSCs) after 1 day. **b** Representative maximum projection images of bins corresponding to (**b**) high RGD, low HAV (outlined blue) and (**c**) low RGD, high HAV (outlined gold) qualitatively show low and high nuclear Sox 9, respectively. **d** Single-cell analysis of nuclear Sox9 fluorescence for these same groups from **b** and **c**. **e** RGD curves at a fixed concentration (light, medium, and dark red correspond to low “L”, medium “M”, and high “H” RGD concentrations, respectively) show a decreasing trend along HAV concentration gradient. In contrast, (**f**) HAV curves at a fixed concentration (light, medium, and dark green correspond to low “L”, medium, “M”, and high “H” HAV concentrations, respectively) show an increasing trend along RGD concentration gradient, with highest values occurring along high HAV concentration curve at a low RGD concentration. *n* > 50 cells in samples shown in **b** and **c**, ***p < 0.001. Scale bars: 50 µm

**Fig. 6:**
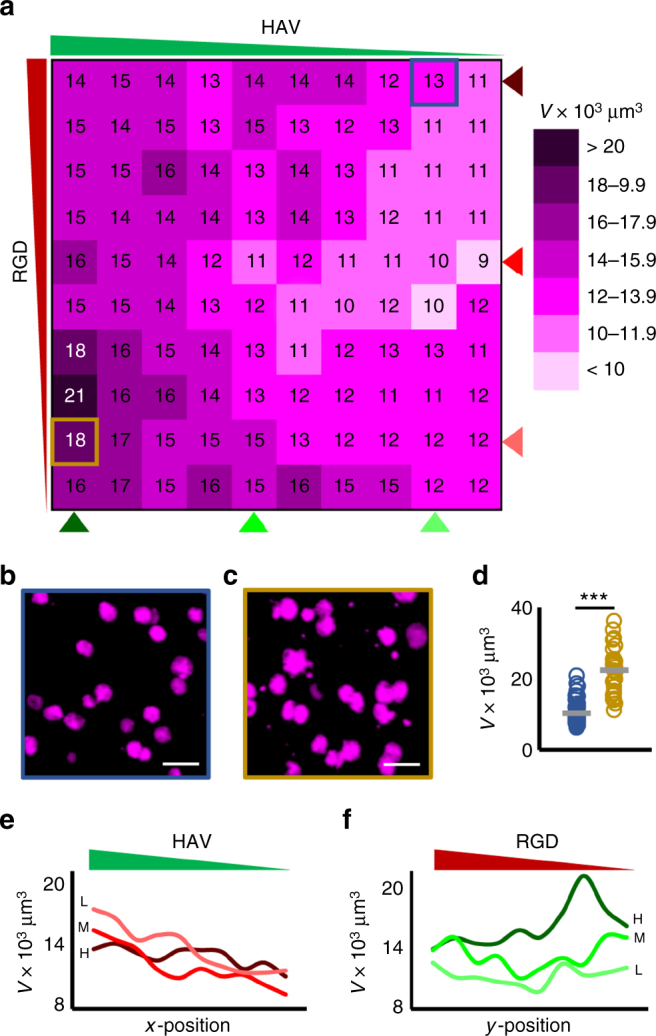
Effects of orthogonal HAV and RGD gradients on aggrecan synthesis. **a** Heat map of synthesized aggrecan volumes across 100 bins (10 by 10 array) for analysis for cultures of mesenchymal stem cells (MSCs) after 7 days. Representative maximum projection images of bins corresponding to **b** high RGD, low HAV (outlined blue) and **c** low RGD, high HAV (outlined gold) qualitatively show lower and higher aggrecan volume, respectively. **d** Single-cell analysis of aggrecan volume for these same groups from **b** and **c**. **e** RGD curves at a constant concentration (light, medium, and dark red correspond to low “L”, medium “M”, and high “H” RGD concentrations, respectively) show a decreasing trend along HAV concentration gradient. In contrast, (**f**) HAV at a constant concentration (light, medium, and dark green correspond to low “L”, medium, “M”, and high “H” HAV concentrations, respectively) show an increasing trend along RGD concentration gradient, with highest aggrecan volume occurring along high HAV concentration curve at a low RGD concentration. *n* > 50 cells in samples shown in **b** and **c**, ***p < 0.001. Scale bars: 50 µm

The influence of combinations of HAV and RGD on MSC chondrogenesis was investigated on single-cell nuclear Sox9 expression and aggrecan synthesis. Briefly, MSCs were photoencapsulated in NorHA hydrogels that were first patterned with an HAV gradient (0–5 mM) in the horizontal direction, followed by an orthogonal RGD gradient (0–5 mM) in the vertical direction, as shown in Fig. 4. Next, after either 1 or 7 days in culture, hydrogels were fixed and stained for nuclear Sox9 or aggrecan, respectively, followed by confocal imaging of the top 100 µm of whole hydrogels. Whole-hydrogel images were then used to analyze discrete regions within the hydrogel that correspond to unique combinations of HAV and RGD peptide concentrations. This was accomplished by discretizing whole hydrogels into 100 bins (10 by 10 array) for analysis, which was then represented as a heat map, as shown for nuclear Sox9 fluorescence (Fig. 5a) or aggrecan volume (Fig. 6a).

Overall observation of whole hydrogel heat maps showed that regions of high RGD and low HAV (i.e., top-right) displayed low nuclear Sox9 levels and low aggrecan volumes, whereas regions of high HAV and low RGD (i.e., bottom-left) displayed significantly higher nuclear Sox9 levels and aggrecan values. Indeed, MSCs stained for Sox9 in an upper-right bin, which corresponds to high RGD and low HAV peptide concentrations, show dim nuclei (Fig. 5b). In contrast, a region representative of cells in a lower-left bin, which corresponds to low RGD and high HAV peptide concentrations, show significantly brighter nuclear Sox9 signal (Fig. 5c). Similar findings were observed for representative cells stained for aggrecan, where a region of high RGD and low HAV (Fig. 6b) displayed significantly lower aggrecan values when compared to a region of low RGD and high HAV (Fig. 6c). Quantification of nuclear Sox9 (Fig. 5d) and synthesized aggrecan (Fig. 6d) further supports these findings. Taken together, these data suggest that regions of high HAV and low RGD are favorable for chondrogenesis.

To further investigate the role of HAV concentration gradients on chondrogenesis, nuclear Sox9 fluorescence values were plotted for MSCs at low, medium, and high RGD concentrations as a function of HAV concentration (Fig. 5e). For all curves at a fixed RGD concentration, highest Sox9 is observed at highest HAV concentration, with the low RGD concentration curve exhibiting the highest starting value. Furthermore, the three curves feature a decreasing trend, with the lowest Sox9 value belonging to the high RGD concentration curve with lowest HAV concentration. An opposite trend was observed when Sox9 values were plotted for MSCs on low, medium, and high HAV concentrations as a function of decreasing RGD concentration (Fig. 5f). Interestingly, these curves show low nuclear Sox9 values in high RGD concentration regions, regardless of HAV concentration. In addition, these curves show a sharp increase in Sox9 fluorescence at an intermediate RGD concentration, with the highest Sox9 fluorescence occurring in the high HAV concentration curve with low RGD concentration (Fig. 5f). Similar trends were observed when the same analysis was conducted for aggrecan synthesis. Aggrecan values plotted for MSCs at constant RGD concentrations along decreasing HAV concentration showed a decreasing trend in synthesized aggrecan (Fig. 6e). Furthermore, curves at a fixed HAV concentration along decreasing RGD concentration showed an increasing trend (Fig. 6f), with the highest aggrecan volume observed on the high HAV concentration curve with a low concentration of RGD (Fig. 6f). In summary, these findings show that generally high HAV with a low amount of RGD results in the highest expression of chondrogenic readouts Sox9 and aggrecan.

### Scaling findings from hydrogel screens to discrete hydrogels

**Fig. 7:**
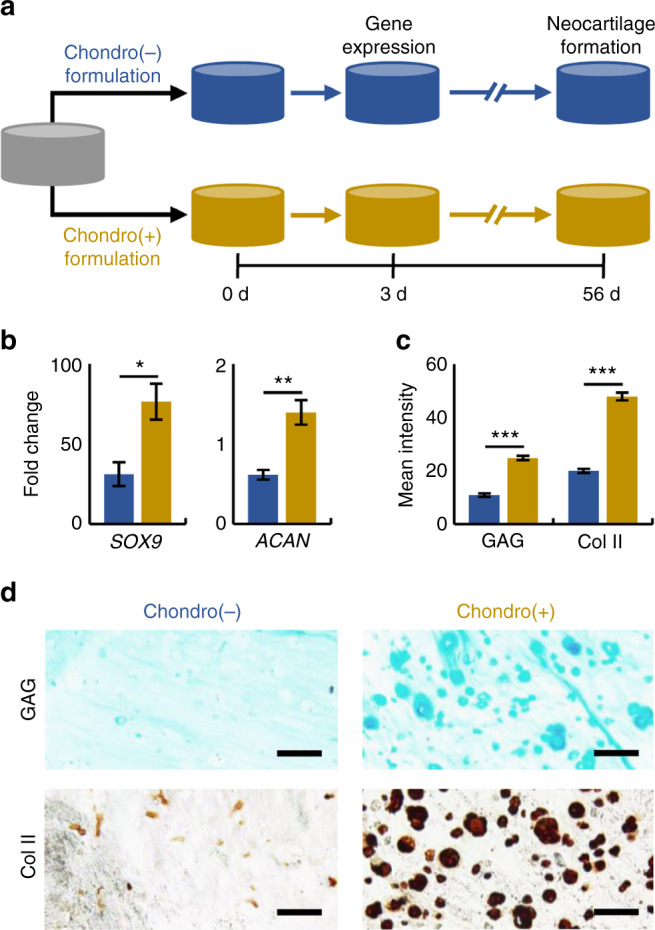
Discrete hydrogels with high HAV and low RGD enhance neocartilage formation by MSCs in vitro. **a** Overview of discrete hydrogel study where mesenchymal stem cells (MSCs) were photoencapsulated in norbornene-modified hyaluronic acid (NorHA) hydrogels and then biofunctionalized with a 5 mM peptide solution consisting of either “Chondro(+)” (4.5 mM HAV and 0.5 mM RGD) or “Chondro(−)” (4.5 mM RGD and 0.5 mM HAV) peptide formulations, and assayed for early (3-day) and long-term (56-day) gene expression and neocartilage formation, respectively. **b** Gene expression of *SOX9* and *ACAN* (aggrecan) after 3 days for MSCs in Chondro(+) and Chondro(−) hydrogels (*n* = 4 hydrogels per group). **c** Quantification and **d** images of histology and immunohistochemistry for glycosaminoglycan (GAG) and type II collagen (Col II) after 56 days (*n* > 50 sections per group). Error bars represent standard error around the mean (s.e.m.); ***p < 0.001, **p < 0.01, *p < 0.05 compared to the Chondro(−) condition. Scale bars: 100 µm

An important step for validating the combinatorial hydrogel platform is to translate the findings to larger constructs that may be useful as therapeutics. To this end, macroscale hydrogels were created using the same multi-step thiol-norbornene UV light-mediated reaction between NorHA and di-thiols (DTT) to form a hydrogel, followed by reacting pendant norbornenes with mono-thiolated peptides to tether biochemical signals to the hydrogels. Specifically, peptide combinations found to induce high (high HAV, low RGD) or low (low HAV, high RGD) single-cell chondrogenic biological readouts (nuclear Sox9 expression, aggrecan volume) were used. It should be noted that both supported chondrogenesis based on their expression of these markers, but their level was altered through local peptide concentration. MSC-laden (20 million cells per ml) NorHA hydrogels were formed on cylindrical molds (5 mm diameter, 1 mm depth) by irradiating with UV light (10 mW/cm^2^) for 10 min. Hydrogels were then incubated with either “Chondro(+)” (4.5 mM HAV and 0.5 mM RGD) or “Chondro(-)” (4.5 mM RGD and 0.5 mM HAV) peptide solutions with 0.05 wt% I2959 in PBS for 30 min and whole hydrogels were irradiated with UV light (5 mW/cm^2^) for 60 s (Supplementary Figure 6). For both the Chondro(+) and Chondro(−) conditions, a total of 5 mM was used so that peptide concentration did not influence the findings. It is also important to note that although screening studies were done at a low density (5 million cells per ml) to allow for single cell analysis, higher seeding densities favor chondrogenesis within hydrogels^36,37^. Therefore, a higher seeding density (20 million cells per ml) was used for these scale-up studies. Chondro(+) and Chondro(−) hydrogels were cultured in chemically-defined medium supplemented with TGF-β3 for either 3 or 56 days and assayed for gene expression and neocartilage formation, respectively (Fig. 7a). The time points chosen were based on previous studies of MSCs encapsulated in hydrogels that showed maximal chondrogenic gene expression in the presence of HAV peptide after 3 days in culture^22^ and significant differences in glycosaminoglycan (GAG) and type II collagen (Col II) content after 56 days in culture^38^.

The fold change of chondrogenic genes *SOX9* and *ACAN* (aggrecan) was significantly higher for MSCs in Chondro(+) hydrogels in comparison to the Chondro(-) group (Fig. 7b). Furthermore, quantification of staining for GAG and Col II was significantly higher in Chondro(+) hydrogels compared to the Chondro(-) condition (Fig. 7c). Representative images of immunohistochemistry staining for GAG and Col II revealed more intense pericellular staining, suggesting more neocartilage matrix formation in Chondro(+) constructs (Fig. 7d). Interestingly, the ECM produced by the cells was exclusive to the pericellular area, which may be due to the high crosslinking density of the 4 wt% hydrogels.

These results indicate that combinatorial hydrogels can be used to screen for cell-material interactions and that the results translate to changes in macroscale hydrogels. In this proof-of-concept study on the platform development, a system that supported chondrogenesis was used with peptides where there was already some understanding of how they may influence these outcomes. This approach allowed development of the system, laying the basis for future cell-hydrogel studies. Although this approach is a great advance beyond iterative studies of individual combinations, these studies are not without their own limitations. One concern for this platform is the influence of paracrine signals from cells in adjacent regions with different biochemical signals on readouts from the screens. Also, since the peptide gradients are continuous, each region investigated is individually heterogeneous. Here, discrete hydrogels show that the cell density and binning resolution used in the combinatorial hydrogel screens is adequate to identify biochemical formulations that induce chondrogenesis and are scalable to larger hydrogel constructs. Additionally, this study was carried out to 56 days, so it is also interesting that the effects from biochemical signals persisted well beyond the duration of the combinatorial screen studies. It would be possible to change parameters such as the speed in which the mask moves to alter the gradient steepness, or perhaps even moving to step-wise changes to alter the biochemical presentation even further.

## Methods

### NorHA and biochemical peptide synthesis

Sodium hyaluronate (Lifecore, 75 kDa) was converted to its tetrabutylammonium salt (HA-TBA) using the Dowex 50 W proton exchange resin, frozen, and lyophilized. HA-TBA carboxylic acid groups were then modified with norbornene groups via amidation with 5-norbornene-2-methylamine, anhydrous dimethyl sulfoxide (DMSO), and benzotriazole-1-yl-oxy-tris-(dimethylamino)-phosphonium hexafluorophosphate (BOP) under nitrogen at room temperature for 2 h. The reaction was quenched with cold water, purified via dialysis (SpectraPor, 6–8 kDa molecular weight cutoff) for 7 days at room temperature, frozen, and lyophilized. The degree of modification was ~57% as measured by ^1^H NMR (Bruker, Supplementary Figure 1).

RGD (GCGYGRGDSPG, 1025.06 g mol^−1^) and HAV (HAVDIGGGC, 869.95 g mol^−1^) peptides with cysteine residues at the C-terminal were obtained from GenScript. Rhodamine-labeled RGD (RhodamineB-GYGRGDSPCG, 1436 g mol^−1^) and FITC-labeled HAV (5(6)-carboxyfluorescein-GHAVDIGGGCG, 1343 g mol^−1^) peptides were synthesized using standard solid state methods, as previously described^39^. Peptides were cleaved in trifluoroacetic acid for 3 to 6 h, precipitated in ether, lyophilized, and stored in −20 °C until use.

### Hydrogel fabrication

Hydrogels were prepared by mixing NorHA macromer, di-thiol crosslinker, and 0.05 wt% photoinitiator Irgacure 2959, (I2959, Ciba), pipetting into PDMS molds (5 × 5 × 0.5 mm^3^). Hydrogel solutions were then photopolymerized with a curing lamp (OmniCure S1500, Excelitas Technologies) with an internal UV filter (320–390 nm) at an intensity of 10 mW/cm^2^ for 10 min. UV intensity was determined using a light meter (YK-35UV, Lutron) with a detector spectrum ranging from 290 to 390 nm, and the intensities used here are within the viability range of previous encapsulated MSC hydrogel studies^40,41^. To create single peptide concentration gradients, NorHA hydrogels were incubated in a 5 mM solution of either mono-thiolated RGD or HAV peptide and 0.05 wt% of photoinitiator I2959 in PBS for 30 min, and exposed to UV light (5 mW/cm^2^) for different times using an opaque sliding mask at a constant rate of 83.3 µm/s. To create orthogonal concentration gradients, after creating an initial HAV gradient, hydrogels were incubated in a 5 mM solution of mono-thiolated RGD peptide and 0.05 wt% of photoinitiator I2959 in PBS for 30 min, and exposed to UV light for different times using an opaque sliding mask at the same rate as before, but in a perpendicular direction. To remove unbound peptide, multiple PBS washes ( > 4 times for 5 min each) were performed following each irradiation step.

### Hydrogel characterization

To characterize the presence of single and orthogonal peptide concentration gradients, gradients were incorporated using rhodamine- and fluorescein-labeled RGD and HAV peptides, respectively. Due to the high fluorescence of the dyes, hydrogels were incubated in 5 mM total peptide solution containing 0.1 mM labeled peptide and 4.9 mM unlabeled peptide. After generating fluorescent gradients, image composites of whole hydrogels were acquired using a Leica TCS SP8 confocal microscope with a motorized *x*–*y* stage. For single gradients, ImageJ software (NIH) was used to divide confocal image composites into 10 evenly-spaced vertical regions, in which the mean intensity for each region was computed and averaged; for orthogonal gradients, images were split into fluorescein-HAV and rhodamine-RGD channels, and then divided into 10 evenly spaced vertical and horizontal regions for mean intensity calculations of HAV and RGD channels, respectively (Supplementary Figure 5).

Hydrogel mechanics were assessed using AFM-based nanoindentation. AFM was used to generate force curves of NorHA hydrogels with either single or orthogonal peptide concentration gradients across a 10 by 10 array using a 25 µm polystyrene spherical probe at 10 μm/s effective indentation rate in PBS (0.03 N m^−1^, Arrow-TL1Au-50, NanoWorld). Indentation moduli were obtained from force-indentation curve data using the elastic Hertz indentation model, assuming a Poisson’s ratio of 0.49 for each force curve of the heat map, as described previously^42^. Effective indentation moduli were then used to construct 10 by 10 heat maps (at least 25 measurements were acquired for each bin), to interpret the data. Values of the averages of individual bins from two hydrogels with s.e.m. are shown in Supplementary Figure 7.

### Cell culture

Human MSCs (Lonza) were expanded to passage 3 in growth media comprised of DMEM with 10% (v/v) FBS (Gibco) and 1% (v/v) penicillin-streptomycin (Invitrogen). All polymers, peptide, and cell culture reagents were either sterile filtered or sterilized via germicidal UV irradiation prior to cell culture. For combinatorial hydrogel screens, hydrogel precursor was prepared by suspending MSCs to a final concentration of 5 million cells per ml in a solution comprised of 4 wt% NorHA macromer and 0.05 wt% I2959 in PBS. Hydrogel precursor was pipetted into 5 × 5 × 0.5 mm^3^ molds, covered with Sigmacote-treated (Sigma) coverslips, and irradiated with UV light (10 mW/cm^2^) for 10 min. For discrete hydrogels, a similar procedure was followed, but the hydrogel precursor solution contained a final MSC concentration of 20 million cells per ml and was pipetted into 5 mm diameter cylindrical molds (depth of 1 mm). After MSC-laden hydrogels were modified with HAV and RGD peptides, hydrogels were cultured in chemically-defined medium (DMEM supplemented with 1% ITS + , 1 mM sodium pyruvate, 40 mg/ml l-proline, 100 nM dexamethasone, 50 µg per ml ascorbic acid 2-phosphate, and 1% penicillin/streptomycin) supplemented with 10 ng/ml TGF-β3. Medium was replaced every 2 days for the duration of the combinatorial hydrogel screen (1- or 7-day) and discrete macroscale hydrogel (3- or 56-day) studies.

### Hydrogel staining and imaging

Cell-hydrogel constructs were prepared for immunocytochemistry by first fixing cells in 10% buffered formalin for 1 h, permeabilizing in Triton X-100 for 30 min, and blocking non-specific binding sites with 3% BSA in PBS for 1 h at room temperature. Next, 1-day constructs were incubated with Sox9 primary antibody (abcam, ab76997, 1:200) and 7-day constructs were incubated with aggrecan primary antibody (abcam, ab3778, 1:50) at 4 °C for 16 h. Hydrogels were then rinsed five times with PBS and incubated with secondary antibody (Invitrogen, AlexaFluor 488 goat anti-mouse, 1:200) for 2 h at room temperature. Samples were then rinsed five times with PBS and incubated with DAPI (Life Technologies, 1:500) for 30 min to visualize nuclei. Using a Leica TCS SP8 confocal microscope with a motorized *x*-*y* stage, high-resolution 3D tile scan images of the top 100 µm of hydrogels were acquired with a step-size of 4 µm, which were used for subsequent analysis.

### Screening analysis

For hydrogels with single biochemical gradients, whole hydrogel confocal image stacks of 1-day Sox9 and 7-day aggrecan stained samples were divided into regions of interest (ROI) consisting of 10 vertical regions (500 µm wide, 5 mm long, *z*-depth 100 µm) in the horizontal direction prior to analysis. For hydrogels with orthogonal biochemical gradients, whole hydrogel confocal image stacks of 1-day Sox9 and 7-day aggrecan stained images were divided into 100 ROIs (500 by 500 µm squares, z-depth 100 µm), and Sox9 nuclei intensity and aggrecan volume were acquired for each bin.

To analyze 1-day ROIs, 3D binary masks of cellular nuclei were generated using Otsu’s intensity-based thresholding method, as done in previous single cell image analysis studies^21,31^. These masks were superimposed to corresponding Sox9 image stacks to acquire nuclear Sox9 intensity on a per-cell basis. For each nucleus, ImageJ’s 3D Objects Counter function was used to calculate volume and total pixel intensity of Sox9 in the corresponding nuclear domains. Sox9 intensity was then reported as total pixel intensity of Sox9 normalized to nuclear volume. To calculate synthesized aggrecan in 7-day ROIs, Otsu’s intensity-based thresholding method was used to generate 3D binary masks of aggrecan, and these masks were subject to ImageJ’s 3D Objects Counter function to calculate aggrecan volume for each cell. For hydrogels with single biochemical gradients, data was reported as the averages of single cells (*n* > 300 per group) across 10 regions in the *x*-direction with s.e.m. shown as error bars. For hydrogels with orthogonal biochemical gradients, data was reported as averages used to construct 10 by 10 heat maps, to interpret the data. Values of the averages of individual bins from two independent biological experiments with s.e.m. can be found in Supplementary Figure 8.

### Gene expression and histological analysis

Samples (*n* = 4) were disrupted in Trizol (Invitrogen) with a tissue homogenizer, and RNA was extracted from these homogenized samples according to manufacturer instructions and subsequently measured with an ND-1000 spectrophotometer (Nanodrop Technologies). Next, 1 µg of RNA from each sample was used for cDNA synthesis using commercial reverse transcriptase (Superscript II, Invitrogen) and oligoDT primer (Invitrogen). Real time polymerase chain reaction (PCR) was performed using an Applied Biosystems 7300 Real-Time PCR system (25 µl reaction volume, 96-well format) with Taqman (5′-nuclease) reactions. Primers and probes specific for glyceraldehyde 3-phosphate dehydrogenase (*GAPDH*), aggrecan (*ACAN*), and *SOX9* are shown in Supplementary Table 1. *GAPDH* was used as the housekeeping gene. Relative gene expression was assessed using the ΔΔ*C*_T_ method, where fold difference is found by the expression $2^{{- \Delta \Delta C}_{\mathrm T}}$.

For histological analysis, constructs were fixed in 10% formalin for 24 h, dehydrated, cleared, embedded in paraffin, and subsequently processed into histological sections (5 µm thick), which were stained for Col II and sulfated GAG. For image analysis, whole images of randomly selected regions of each sample were first converted to 8-bit and then inverted. Mean staining intensity within these randomly placed frames was measured using ImageJ.

### Statistical analysis

All images and data are representative of the results of two or more independent biological experiments. Statistical significance was determined using one-way ANOVA with Tukey’s post hoc test using Excel software with Daniel’s XL Toolbox add-in. Differences between culture conditions were considered significant for *p*-values <0.05, and error bars indicate standard error around the mean (s.e.m.).

### Data availability

All relevant data are available upon request from the authors.

## Electronic supplementary material

Supplementary Information
