# Supplementary material for: Combinatorial hydrogels with biochemical gradients for screening 3D cellular microenvironments
Source: Nat Commun. 2018 Feb 9;9:614. doi: 10.1038/s41467-018-03021-5 (PMC5807520; doi:10.1038/s41467-018-03021-5)
Supplement: Supplementary file 1 — Supplementary Information [file 41467_2018_3021_MOESM1_ESM.pdf]

Supplementary Table 1: Primer and probe sequences for quantitative PCR. Sequences related to Sox9 gene are proprietary to Applied Biosystems Inc. and not disclosed.

Gene	Forward Primer	Reverse Primer	Probe
GAPDH	AGGGCTGCTTTTAACTCTGGTAAA	GAATTTGCCATGGGTGGAAT	CCTCAACTACATGGTTTAC
ACAN	TCGAGGACAGCGAGGCC	TCGAGGGTGTAGCGTGTAGAGA	ATGGAACACGATGCCTTTCACCACGA

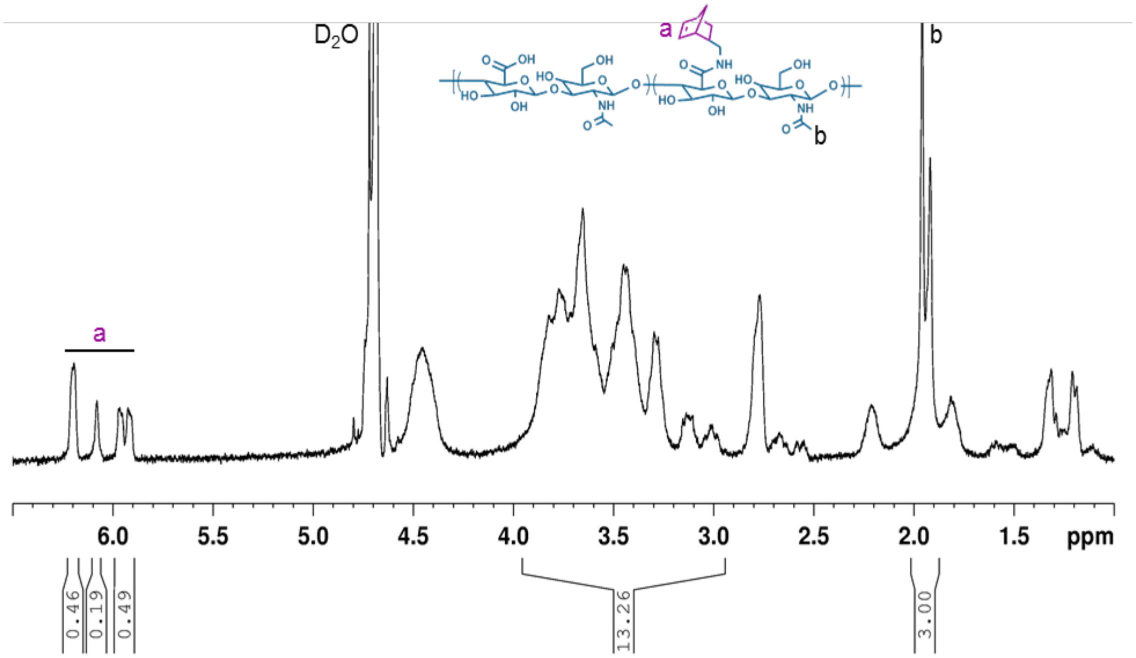

Supplementary Figure 1: ^{1}H NMR spectrum for norbornene-modified hyaluronic acid (NorHA). Spectrum indicates ~57% modification of HA repeat units with norbornene groups.

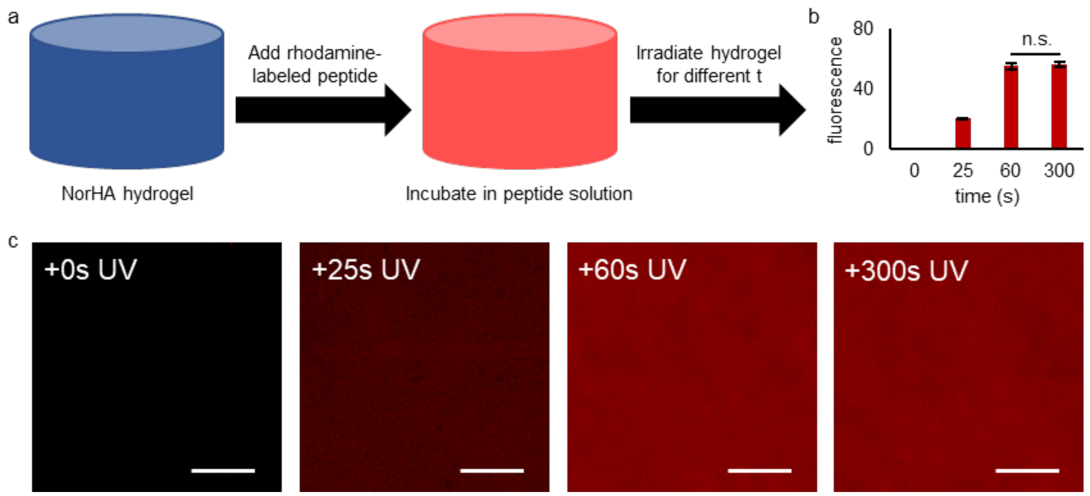

Supplementary Figure 2: Hydrogel fluorescence after introduction of fluorescent peptides. (a) Schematic of the incubation of norbornene-modified hyaluronic acid (NorHA) hydrogels in solution of rhodamine-labeled mono-thiolated RGD peptide (5 mM) and UV irradiation (5 mW per cm^2) for various times. (b) Quantification and (c) representative maximum projection images of fluorescence of hydrogels after irradiation with peptides for various times. The signal intensity increased with irradiation time up to 60 seconds. Error bars represent standard error around the mean (s.e.m.); scale bars: 250 μm; n.s.: no significance between groups; n = 9 measurements per group.

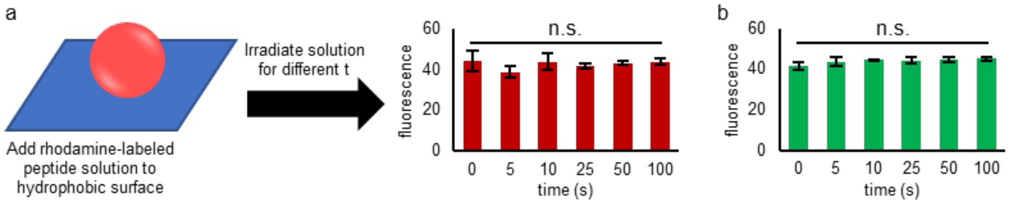

Supplementary Figure 3: Peptide fluorescence with UV irradiation. (a) Schematic of the irradiation of rhodamine-labeled RGD peptide solutions (5 mM total peptide concentration: 4.9 mM unlabeled RGD and 0.1 mM rhodamine-labeled RGD) with UV light (10 mW per cm^2) for various times (0 to 100 seconds). Fluorescence of rhodamine peptide solutions shows no change in fluorescence from UV irradiation. (b) Irradiation scheme presented in (a) was also performed on fluorescein-labeled HAV peptide solutions (5 mM total peptide concentration: 4.9 mM unlabeled HAV and 0.1 mM fluorescein-labeled RGD). Fluorescence of fluorescein peptide solutions shows no change in fluorescence from UV irradiation. Error bars represent standard error around the mean (s.e.m.); n.s.: no significance between groups; n = 9 measurements per group.

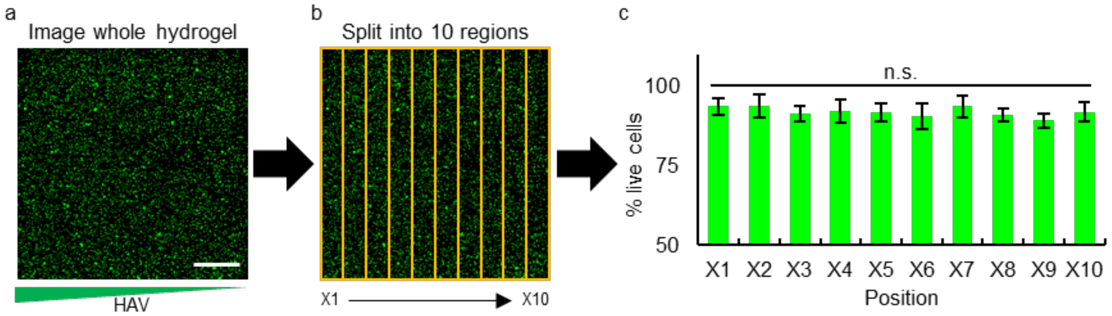

Supplementary Figure 4: 7-day viability of MSCs in hydrogels with HAV peptide gradients. (a) Maximum projection image of the top 100 μm of whole hydrogels 7 days after the encapsulation of mesenchymal stem cells (MSCs) and staining with live/dead assay. (b) Images and (c) quantification of cell viability across the sample when split into 10 equal regions. These results indicate high viability and no decrease in viability with changes in HAV concentration. Error bars represent standard error around the mean (s.e.m.); scale bar: 1 mm; n.s.: no significance between groups; n > 1000 cells per zone.

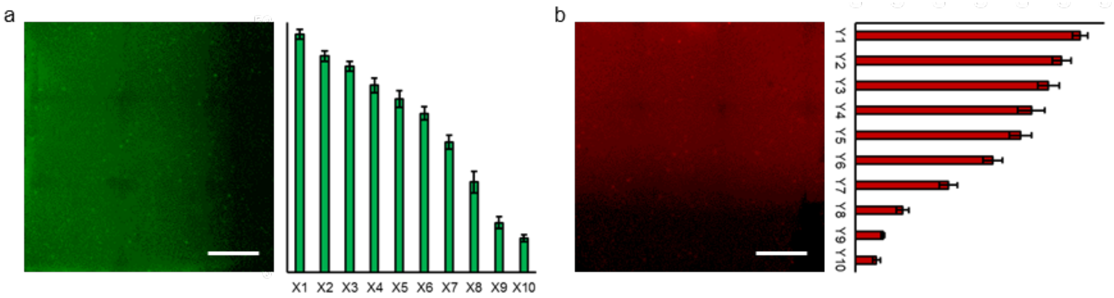

Supplementary Figure 5: Hydrogels with orthogonal biochemical gradients. Imaging and quantification of a single sample separated into (a) horizontal (X1 to X10) fluorescein-labeled HAV and (b) vertical (Y1 to Y10) rhodamine-labeled RGD orthogonal gradients. Error bars represent standard error around the mean (s.e.m.); scale bars: 1 mm; n = 8 measurements per zone.

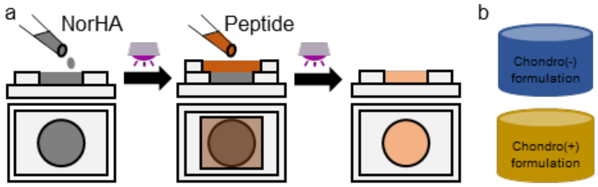

Supplementary Figure 6: Scheme for fabricating discrete NorHA hydrogels. (a) To form hydrogels, norbornene-modified hyaluronic acid (NorHA) solution (NorHA macromer, I2959 photoinitiator, di-thiol (DTT) crosslinker, 20 million mesenchymal stem cells (MSCs) per ml) was added to cylindrical molds (5 mm diameter, 1 mm depth) and irradiated with UV light (10 mW per cm^2) for 10 min. Hydrogels were then incubated in peptide formulations for 30 min and irradiated with UV light (5 mW per cm^2) for 60 seconds. The peptide formulations used were either (b) "Chondro(-)" (4.5 mM HAV and 0.5 mM RGD) or "Chondro(+)" (4.5 mM HAV and 0.5 mM RGD) with 0.05 wt% photoinitiator (I2959) in PBS.

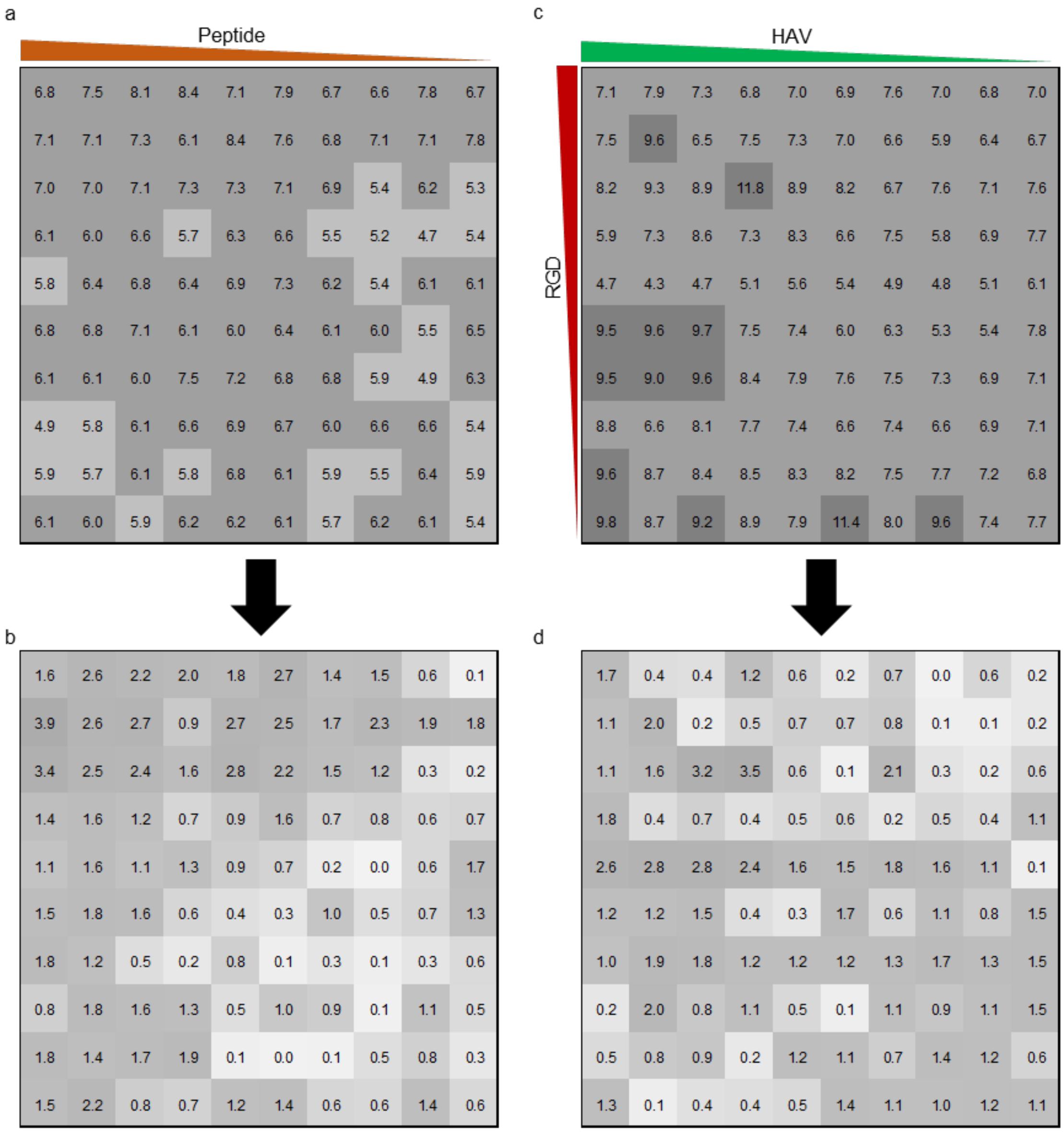

Supplementary Figure 7: Atomic force microscopy (AFM) analysis of hydrogels. (a) Average AFM measurements (n > 25 measurements per bin) of hydrogels exposed to single biochemical gradient (0 to 5 mM), and (b) standard error around the mean (s.e.m.) measurements from two different hydrogels. (c) Average AFM measurements (n > 25 measurements per bin) of hydrogels exposed to orthogonal biochemical gradients, and (d) s.e.m. measurements from two different hydrogels. Units: kPa.

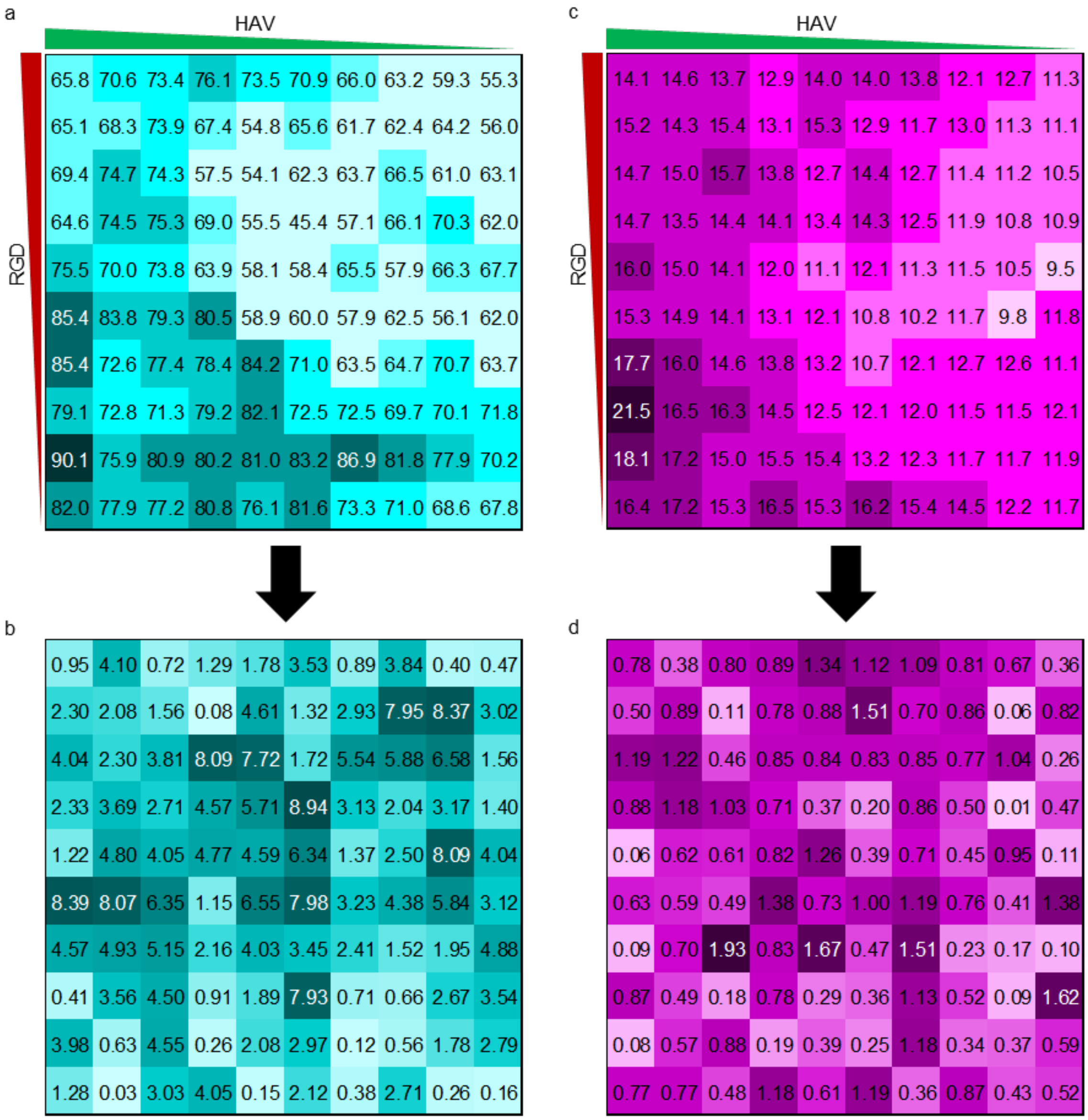

Supplementary Figure 8: Analysis of chondrogenic biological readouts. (a) Average nuclear Sox9 fluorescence measurements (n > 50 cells per bin) of mesenchymal stem cells (MSCs) exposed to orthogonal biochemical gradients, and (b) standard error around the mean (s.e.m.) measurements from two different hydrogels. (c) Average aggrecan volume (x10^3 μm^3) measurements (n > 50 cells per bin) of MSCs exposed to orthogonal biochemical gradients, and (d) s.e.m. measurements from two different hydrogels.
